# CO-CREATION-HF protocol: clinical trial to evaluate the impact of a comprehensive and hybrid cardiac rehabilitation model on patients with heart failure

**DOI:** 10.3389/fcvm.2024.1427544

**Published:** 2024-11-14

**Authors:** Pamela Seron, Daniela Gómez-Pérez, Edgardo Opazo-Díaz, Maria Jose Oliveros, Maria Francisca Contreras, Alejandra Salinas, Omar Andrade-Mayorga, Gabriel Nasri Marzuca-Nassr, Kathleen Saavedra, Cinthya Espejo, Sergio Muñoz, Fernando Lanas, Sherry L. Grace

**Affiliations:** ^1^ Dpto. Ciencias de la Rehabilitación, Facultad de Medicina, Universidad de La Frontera, Temuco, Chile; ^2^ Dpto. de Psicología, Facultad de Educación, Ciencias Sociales y Humanidades, Universidad de La Frontera, Temuco, Chile; ^3^ Dpto. de Kinesiología, Facultad de Medicina, Universidad de Chile, Santiago, Chile; ^4^ Dpto. Medicina Interna, Facultad de Medicina, Universidad de La Frontera, Temuco, Chile; ^5^ Dpto. Ciencias Preclínicas, Facultad de Medicina, Universidad de La Frontera, Temuco, Chile; ^6^ Dpto. Ciencias Básicas, Facultad de Medicina, Universidad de La Frontera, Temuco, Chile; ^7^ Dpto. Salud Pública, Facultad de Medicina, Universidad de La Frontera, Temuco, Chile; ^8^ Faculty of Health, York University, Toronto, ON, Canada; ^9^ KITE Research Institute, University Health Network, Toronto, ON, Canada

**Keywords:** cardiac rehabilitation, exercise, heart failure, telerehabilitation, adherence, psychosocial support

## Abstract

**Introduction:**

Comprehensive, hybrid cardiac rehabilitation (CR) models have been scantly investigated in heart failure (HF) populations, particularly in low-resource settings. CO-CREATION-HF aims to evaluate the effectiveness of such a model compared to supervised exercise alone.

**Methods and analysis:**

A 2 parallel-arm, multi-center randomized clinical superiority trial will be conducted with blinded outcome assessment. 152 HF patients (NYHA class II or III) will be recruited consecutively, and randomly assigned using permuted blocks; allocation will be concealed. The 12-week intervention will include evaluation, medical and nurse management, aerobic interval training, resistance exercise training, psychosocial support, and education. These will initially be delivered in a center, transitioning to home in 4 stages. Controls will receive similar management, but face-to-face continuous aerobic exercise sessions and resistance exercises. The main outcomes are cardiorespiratory fitness (VO_2_ max), functional capacity (m from 6 MWT), and quality of life (Minnesota Living with Heart Failure Questionnaire). Program adherence and completion, NT-proBNP, functioning, all-cause and HF-specific mortality and hospitalization, muscle strength, adverse events and cost will be secondary outcomes. These will be measured at baseline, end of intervention, and 12-month follow-up. The sample size was calculated considering 90% power, a significance level of 5%, a between-group difference equivalent to 1/2 MET, and a 10% potential loss to follow-up. Intention-to-treat analysis will be considered. Between-group differences will be assessed using Student's *t*-tests or *Z*-tests along with 95% confidence intervals, and the rate ratio will be computed to compare mortality.

**Ethics and dissemination:**

The study protocol and the Informed Consent form were approved by Ethical Committees at the Universidad de La Frontera (No. 081-23) and each center participating. Research findings will be disseminated to the scientific community and will be shared with relevant stakeholder groups and policy-makers. Finally, investigators shall reach HF patients via various dissemination channels such as social media.

**Clinical Trial Registration:**

clinicaltrials.gov, identifier (NCT06313684).

## 1 Introduction

Heart failure (HF) is characterized by symptoms and signs such as breathlessness, ankle swelling, and fatigue that result from structural or functional impairment of ventricular filling or ejection of blood (1). HF is a growing global health challenge, with a substantial burden due to low exercise tolerance, poor health-related quality of life (HRQoL), as well as high risk of mortality and hospital admission, which all culminate in high use of healthcare resources and costs (2). Indeed, HF is the leading cause of hospitalizations in people >65 years (3).

Although HF incidence has remained stable or has even declined, prevalence is increasing due to the aging of the population. The age-standardized prevalence of HF varies substantially across regions, ranging from 498 (Southe3ast Asia) to 1,196 (Central Europe) per 100,000, correlating with the sociodemographic development of countries, as it does with the years lived with HF disability. In the Latin American low and middle-income context, high age-standardized rates of prevalent cases are also found, particularly in Central countries in the region, such as Nicaragua with 912.4 and Venezuela with 991 (4).

Several clinical guidelines have been developed to support optimal treatment and monitoring of patients with HF. HF has no definitive cure, and therefore, recommendations are aimed at controlling symptoms, improving functional capacity, optimizing quality of life and prolonging patient survival. Self-management of multiple behaviors is key to preventing acute exacerbations and hospitalizations.

In particular, clinical guidelines internationally recommend exercise or exercise-based cardiac rehabilitation (CR) as a non-pharmacological intervention for HF, but they do so weakly primarily due to the low or moderate certainty of the evidence (1, 5, 6). The Cochrane review of exercise-based CR for HF Patients updated in 2019 (7) which included 44 randomized clinical trials (RCTs), plus additional sequential analysis (8), concluded that compared to no exercise, continuous aerobic exercise appears to have no impact on short-term mortality, probably reduces the risk of hospitalization, and would improve HRQoL. It is important to note that most of the included trials are from “Western” high-resource settings and tested exercise only, with only 3 RCTs in low or middle-income countries and 12 RCTs incorporating other CR components such as education. Comprehensive CR could result in more favorable outcomes, given patients also receive education and psychosocial counseling or support so they understand the importance of self-management via exercise and other health behaviors and can overcome barriers to adherence to these behaviors. Other factors that can lead to disease progression are also addressed in comprehensive CR, such as diet, medication adherence and tobacco cessation where applicable.

The HF-ACTION trial (9) was influential in the Cochrane review because it included more than 1,000 patients per arm, impacting the current guideline recommendation for exercise in patients with HF. Based on this trial—conducted in the United States, Canada, and France– the authors concluded that exercise training was associated with modestly-significant reductions in both all-cause mortality or hospitalization and cardiovascular mortality or HF hospitalization. However, there were some limitations of HF-ACTION, including that it was not pragmatic in its’ approach as patients were provided with special equipment to exercise at home, limiting generalizability especially in low-income settings; as well as the low adherence to the intervention (30%), which is also a problem in routine clinical practice. This latter aspect is fundamental, as patients with HF experience considerable fatigue and breathlessness, resulting in exercise intolerance, so it is necessary to tailor exercise prescriptions individually and incorporate interventions that support adherence. Unfortunately, however, most patients with HF do not even access CR (10), underscoring the challenges in delivering CR for these patients.

Also concerning the issue of the degree of CR impact in HF populations, emerging evidence has supported the benefits of CR of a minimum 3 months (11), as well as with higher-intensity exercise (12), including interval training (13). Indeed, the greater the dose of CR, the lower the mortality, among other benefits (14). However, many trials and real-world CR models often do not offer CR of sufficient “dose”/sessions to realize the significant benefits (15). In low-resource settings, patients receive even fewer CR sessions. Interval training may be of particular benefit in HF because patients can reach higher exercise intensities, optimizing impact of each session attended (16, 17).

An approach to address low adherence for HF is offering home-based delivery (18–20) or hybrid models (21–23) vs. the traditional center-based models. Specifically, hybrid CR, defined as a combination of center-based and home-based, has been shown to improve physical function, exercise capacity and HRQoL in patients with HF when compared with usual care. They have also been shown to be safe in this population (19). However, more studies are needed to clarify if hybrid CR produces superior outcomes than traditional center-based models, and also to better define the role that technology should play in non-supervised CR for HF (22).

**Figure 1:**
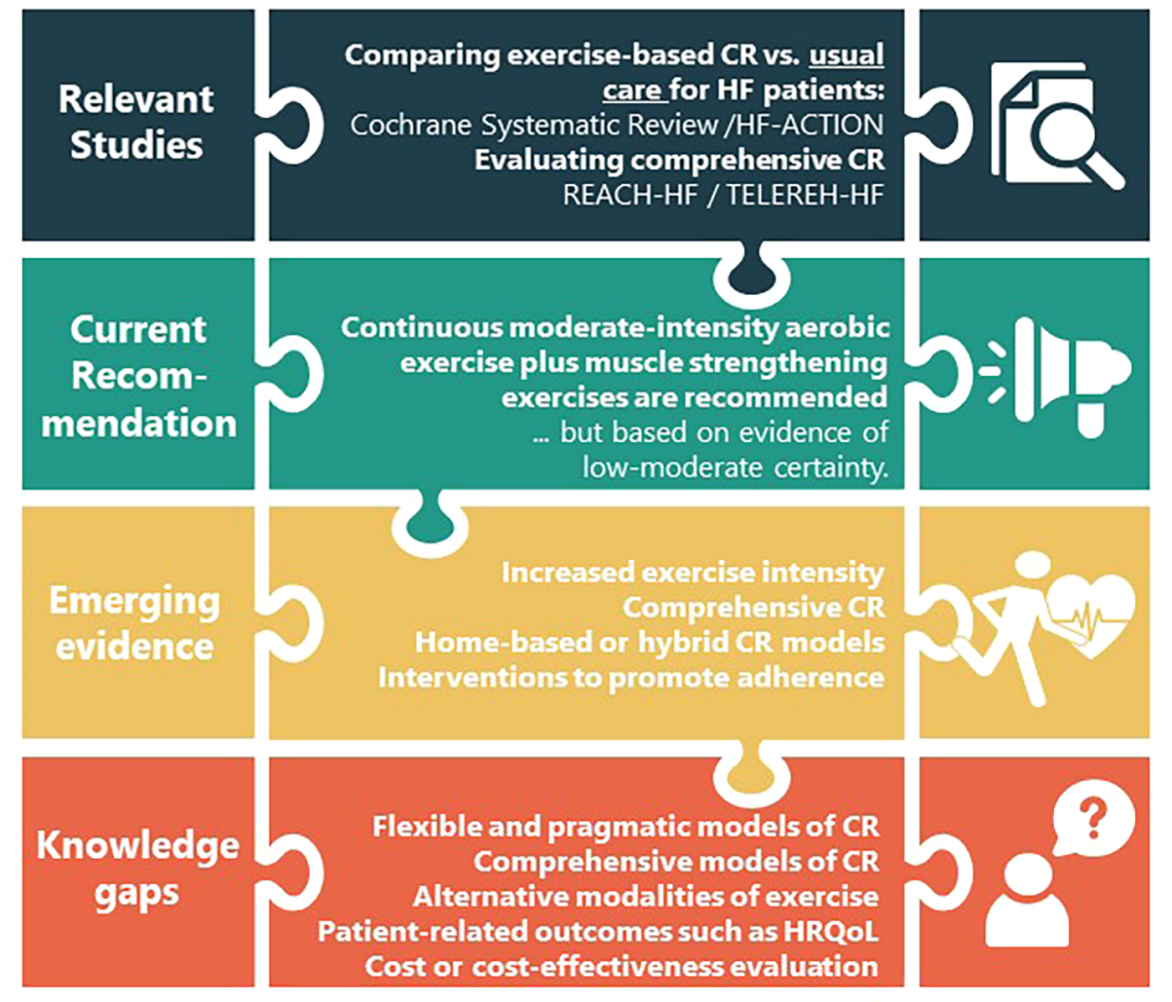
Summary of the scientific novelty of the trial about the heart failure literature.

In light of the available evidence, where the emphasis has been on conventional exercise (basically continuous, supervised aerobic exercise) with little success (probably due to lack of adherence) and recalling the definition of CR as a multi-component intervention, it is time to investigate a comprehensive HF-tailored CR model pragmatically-delivered for low-resource settings addressing the knowledge gaps identified, to achieve optimal outcomes in this challenging population in great need. Indeed, there have been very few clinical trials evaluating the effectiveness of comprehensive hybrid CR in HF patients, such as the REACH-HF (Rehabilitation EnAblement in CHronic Heart Failure) (23) and TELEREH-HF (The Telerehabilitation in Heart Failure Patients) (24) studies in high-resource settings. Figure 1 shows the state of the art and the knowledge gaps that triggered this proposal.

The CO-CREATION-HF (Comprehensive Hybrid Cardiac Rehabilitation Trial to Assess Impact on Heart Failure Patients) trial aims to evaluate the effectiveness of comprehensive hybrid (including the use of mobile phones) CR compared to a supervised exercise-based intervention alone on maximum cardiorespiratory capacity, functional capacity, and HRQoL. Secondarily, effectiveness for program adherence and completion, pro-B-type natriuretic Peptide (pro-BNP), functioning, mortality and hospital admission, skeletal muscle strength, exercise-related adverse events, and costs will also be compared.

## 2 Methods and analysis

### 2.1 Design

A pragmatic, multi-center, 2-parallel arm randomized clinical trial with blinded outcome assessment will be conducted. In this study, a hypothesis of superiority is tested (i.e., a comprehensive and hybrid CR program produces better results than a conventional exercise-based supervised program). This trial will be pragmatic in that it examines the outcomes of the experimental intervention compared with a control group under circumstances that closely approximate the real world, in a low-resource setting. Patients will be recruited in 4 health centers in Chile. It is not possible to double-blind in a CR trial, but personnel undertaking outcome assessment will be blinded to group allocation. Finally, the randomization process (concealed allocation 1:1) will allow balancing in both groups of all known and unknown sociodemographic and clinical characteristics that could confound the association between the intervention and results.

**Figure 2:**
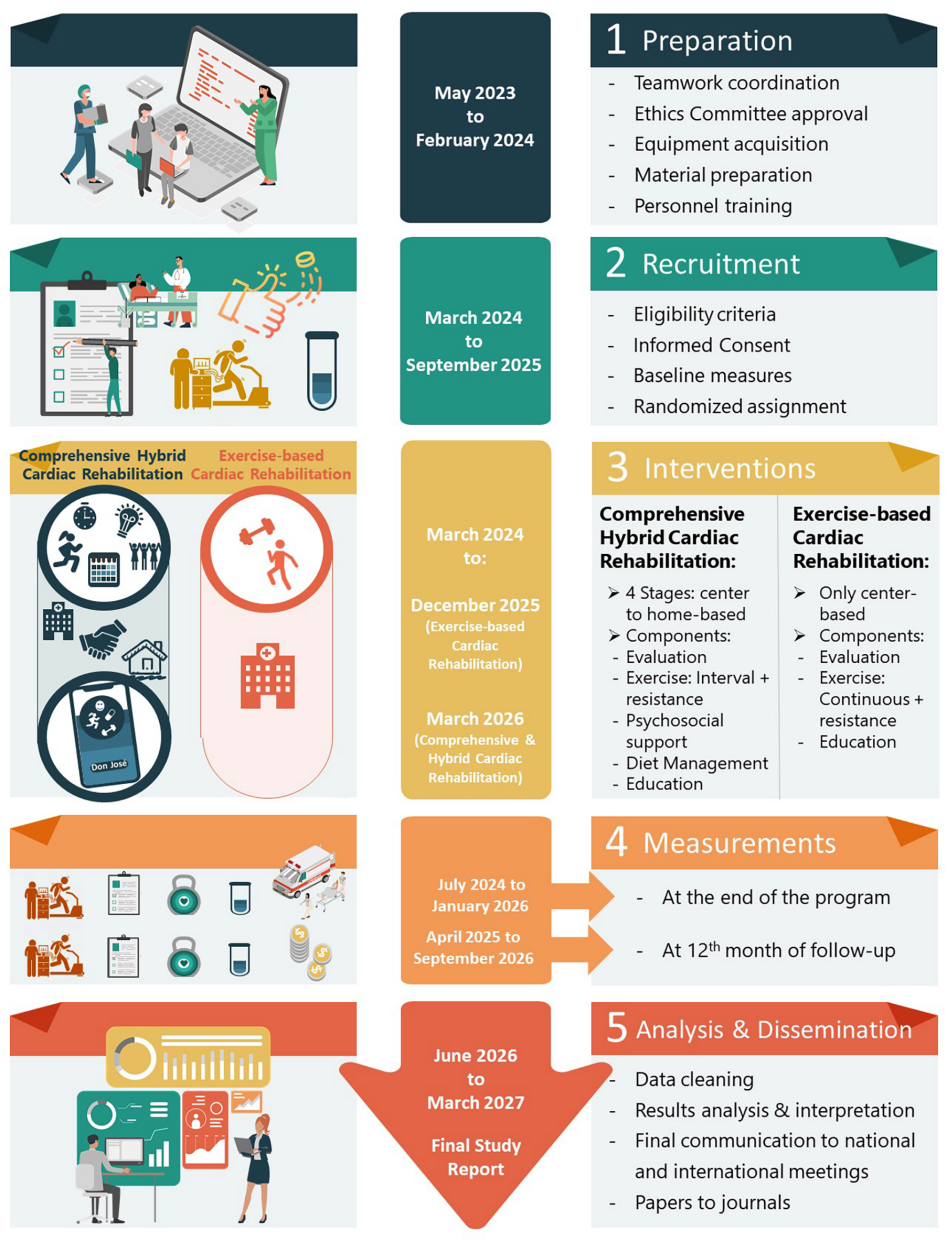
Flowchart and timeline for activities in the CO-CREATION-HF trial.

The trial protocol is registered in clinicaltrials.gov (number NCT06313684). It will be reported in accordance with the CONSORT statement (25), including the extensions for non-pharmacologic treatment interventions, and pragmatic trials. A diagram of the trial showing activities in detail is presented in Figure 2.

### 2.2 Study population

The reference population is chronic HF patients. The accessible population is patients that attend one of the 4 Chilean tertiary hospitals involved in the study: Complejo Hospitalario San José, Hospital Clínico de la Universidad de Chile, Hospital Clínico San Borja Arriarán y Hospital Dr. Hernán Henriquez Aravena. The sampling will be non-probabilistic of consecutive cases, with a sample of patients in the above centers meeting specific eligibility criteria presented in Table 1.

**Table 1:** Eligibility criteria for CO-CREATION-HF trial.

Inclusion Criteria	Exclusion criteria
– Adult patients with HF (HFrEF, HFmrEF, and HFpEF) of New York Heart Association functional class II or III (26) – Meets HF diagnostic criteria of Guidelines (1, 5) • HFrEF: Symptoms/signs of HF and ejection fraction ≤ 40%. • HFmrEF: Symptoms/signs of HF and ejection fraction 41%–49%. • HFpEF: Symptoms/signs of HF, ejection fraction ≥ 50% and objective evidence of cardiac structural and/or functional abnormalities consistent with the presence of left ventricular diastolic dysfunction/raised LV filling pressures, including raised natriuretic peptides. – On maximum tolerated medical therapy available according to current guidelines (5, 27, 28) – Deemed by the treating physician as stable for at least 1 month. – Able to attend the health center three times a week for the first month, and twice a week for the 2nd and 3rd months – Owns a mobile phone – Patient consents to participate in the study by signing an informed consent form.	– Chronic kidney disease with glomerular filtration rate < 20 ml/min. – Decompensated thyroid disease. – End-stage liver failure or Child Pugh C. – Cardiac device (implantable defibrillator or cardiac resynchronization) or cardiac surgery (revascularization or valve procedure) in the previous month or planned in the next 3 months. – Patients with dyspnea predominantly of non-cardiac cause (e.g., decompensated COPD). – Atrial fibrillation with a heart rate greater than 90 beats per minute at rest. – Active neoplasm with life expectancy <2 years. – Inclusion in another interventional study. – Explicit contraindication to performing exercise based on the American College of Sports Medicine guidelines (29). – Comorbidities such as dementia, blindness, deafness, serious mental illness, or frailty (defined as dependent according to EMPAM classification) the precludes the patient from engaging in a CR program. – Musculoskeletal or neurological disease that precludes the patient from performing exercise.

HF, heart failure; HFrEF, heart failure with reduced ejection fraction; HFmrEF, heart failure with mid-ranged ejection fraction; HFpEF, heart failure with preserved ejection fraction; COPD, chronic obstructive pulmonary disease; EMPAM, examen médico preventivo del adulto mayor [preventive health examination of the older adult]; CR, cardiac rehabilitation.

### 2.3 Sample size

One hundred and thirty-eight patients (69 per group) are required to have a 90% chance of detecting as significant at the 5% level, a difference in the primary outcome measure of maximum cardiorespiratory capacity (VO_2_ max), assuming 15 ml/Kg/min in the control group and 16.75 ml/Kg/min in the experimental group at intervention end. The VO_2_ max assumed in the control group is that achieved by the experimental group with exercise-based CR in the HF-ACTION trial (9), with a standard deviation of 3.15 calculated with the range rule. The VO_2_ max in the experimental group was assumed with an intermediate minimal important difference of 1/2 MET (1.75 ml/kg/min) (30, 31). If a potential 10% loss of follow-up is added, results in 152 patients (76 per group). Sample sizes were also calculated for the other two primary outcomes, functional capacity, and HRQoL, with fewer patients needed to test the underlying hypothesis.

### 2.4 Recruitment and randomization

Patients will be invited to participate in one of two ways: at the routine consultation of the HF polyclinic in each hospital, or through a telephone call by the nurse in charge of the HF census operating in two hospitals. All information about the study will be delivered by a nurse, physician or physiotherapist in charge of this task exclusively, and who will be unaware of the randomization sequence. If patients consent to participate in the study, an initial evaluation will be performed to collect baseline sociodemographic as well as clinical characteristics and measures.

Assignment to the experimental or control group will be by permuted blocked randomization and conducted by an independent researcher. Concealment of the assignment will be preserved through the functionality available in REDCap© (32), and will only be known to the therapist who receives the patient in the CR program. The core components for both arms in the study are presented in Table 2.

**Table 2:** Core cardiac rehabilitation components for experimental and control programs.

	Comprehensive hybrid CR	Supervised exercise-based CR
Initial Assessment	Includes all those necessary to prescribe exercise training, as well as dietary and adherence promotion interventions. – Functional capacity with 6 MWT – Assessment of skeletal muscle strength, flexibility, and balance. – Assessment of functioning – Assessment of dietary habits – Assessment of self-efficacy and barriers to CR adherence. – Screening for depression.	Includes all those necessary to plan exercise training, as usual: – Functional capacity with 6 MWT. – Assessment of skeletal muscle strength.
Continuous evaluation:	Evaluations during the course of the program will be carried out to inform the progression through the CR stages, the intensity of training and education needs.	Not applicable.
Medical and nurse clinical management:	Performed following the recommendations in the guideline update of the American and European societies (1, 28). 25	
Exercise	High-intensity interval training prescribed by physical therapist: – According to an adapted Wisloff protocol (33). – Borg scale to monitor intensity (34). – Training 3 times per week. Resistance exercises training: – With TheraBand OMNI-Resistance exercise bands. – Intensity will be based on participant's subjective perception, according to the perceived exertion scale (70%–80% 1 RM) – Frequency at least twice a week.	Moderate-intensity continuous exercise prescribed by physical therapist: – The intensity will be moderate, as tolerated – Borg scale to monitor intensity (34). – Frequency of training of 1 to 3 times per week, until completed 20 sessions in 10–12 weeks. Resistance exercices. – With available materials. – Moderate intensity – 1–2 times a week.
	Exercise volumes (interval plus resistance and continuous plus resistance) shall be equivalent between the experimental and control groups.	
Psychosocial support:	Based on social-cognitive theory (35) and delivered by a trained professional (occupational therapist or nurse), with self-efficacy as a focus, it will include: – Two face-to-face sessions in the first stage of the program: Education, self-monitoring and motivational interview. – One face-to-face session in the second stage of the program: promoting behavior changes by self-control and self-monitoring – Regular communication with patients will be supported by the use of mobile devices during the third and fourth stages of program. – Referral to a specialist will be considered if depression is detected in the screening.	Not applicable
Diet management	– Dietary plan supported by a nutritionist will be made together with the patient.	Not applicable

CR, cardiac rehabilitation; RM, repetition maximum; 6 MWT, 6 min walk test.

### 2.5 Experimental group

The participants randomly assigned to the experimental group will participate in a comprehensive, hybrid CR program, completed in 4 stages as presented in Table 3, and guided by the following 5 principles:

– Pragmatic: program implementation will be carried out as close to real-life as possible, that is to say, with the resources that a traditional program has in Chile, while adhering to minimum requirements and ensuring fidelity.
– Interdisciplinarity: The design and implementation of the CR intervention will be carried out by an interdisciplinary team involving at least a physician, a nurse, a physiotherapist, a nutritionist, and a psychologist.
– Progression: mainly of the intensity and frequency of exercise (36). All other interventions and the transition from the center-based to the home-based phase will have a pre-established progression.
– Reproducibility: individualized exercise prescription, progression and transition to home-based CR will be explicit and reported.
– Accompaniment: this is a key principle for monitoring progress and reinforcing interventions designed to promote adherence to interventions.

**Table 3:** Comprehensive hybrid cardiac rehabilitation program protocol for the experimental group.

Stage	Characteristics	Duration
1. Fully supervised and center-based	– Supervised center-based sessions of high intensity interval training and resistance exercise. – Emphasis on education in the performance of the exercise, with the aim that it can be done independently in the future. – Intervention to promote adherence and diet management with two face-to-face sessions.	– Begins on admission to the program, and lasts for 4 weeks or earlier if the patient has learned to perform the strength exercises on their own, or later if the patient needs make-up some missed supervised sessions.
2. Transition to home-based	– Supervised center-based high intensity interval training with less frequency. – Reviewing the resistance exercises that are being done at home. – Intervention to promote adherence and heart-healthy diet will be monitored remotely in a weekly communication.	– Beginning at the end of stage 1 and continues through to 8 weeks.
3. Home-based with monitoring	– Training is carried out at home and is monitored through a call at least once a week by the physiotherapist. – Psychosocial support and adherence to exercise, diet and medication will be promoted through phone calls once a week. – If necessary, a face-to-face session would be offered at this stage to reinforce any identified challenges and for progress evaluation.	– This stage will last 4 weeks or more, through to 12 weeks.
4. Home-based without monitoring	– The patient will do the training at home unsupervised. – Phone calls will be held once every two weeks for two months, and then once a month through to the 6 months. – These calls will be designed to reinforce adherence and resolve patient questions regarding exercise, diet, or medication.	– From week 12 to week 24.

**Figure 3:**
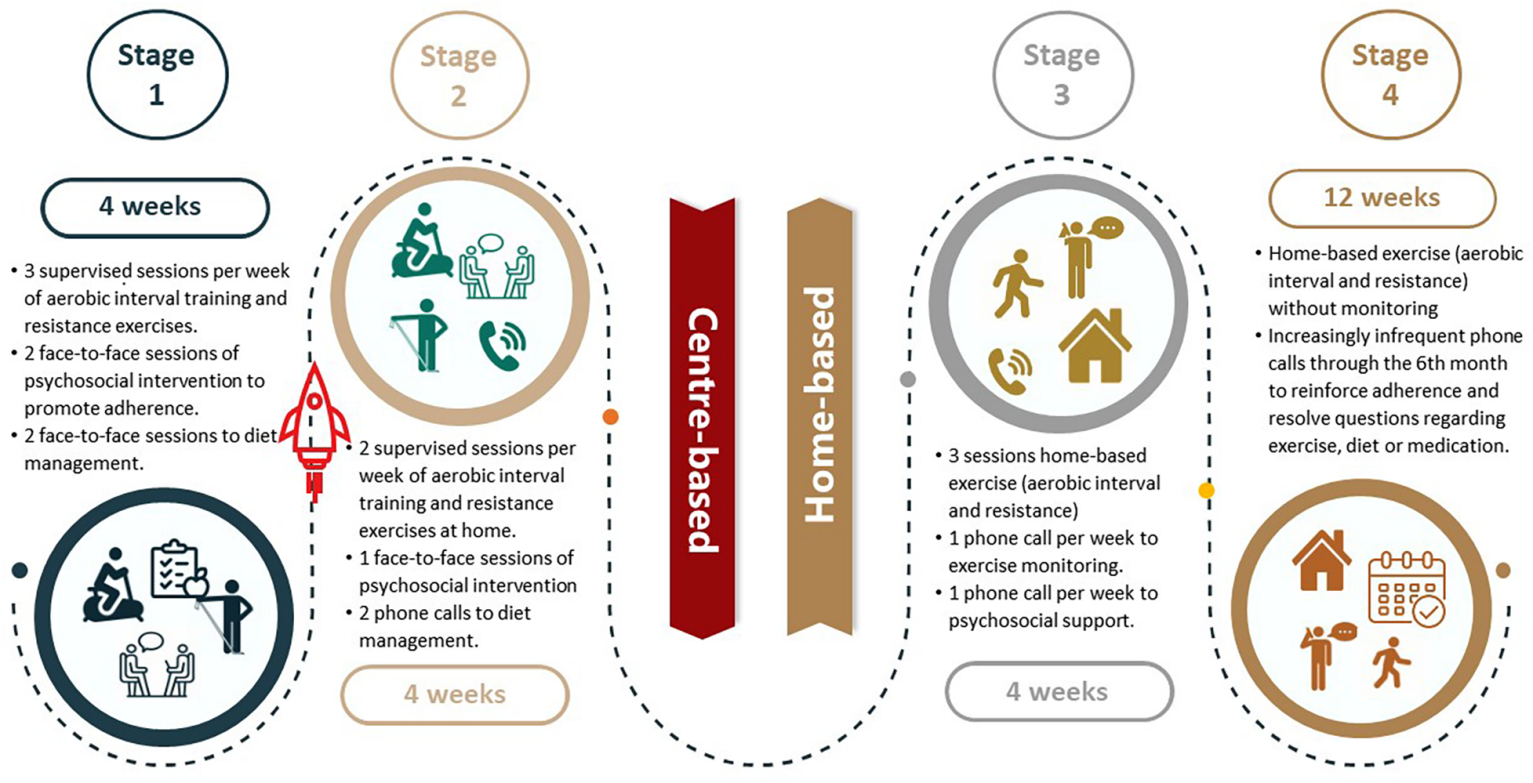
Schematic overview of the four stages of the experimental intervention.

The comprehensive, hybrid CR program will begin with an initial evaluation that includes all necessary assessments to prescribe exercise training as well as dietary and adherence promotion interventions (Table 3, Figure 3). The exercise component includes aerobic interval training and resistance exercises. The center-based training will be performed on an ergometer bike; as the CR program progresses, the training will be adapted to a modality that can be undertaken by the patient at home, such as using of a footstool, static bike if it is available, or walking outdoors.

For the aerobic interval training, the Wisløff protocol (33), with long work intervals, will be adapted. Each session will consist of a 10 min warm-up, followed by 20 min of high-intensity interval training, with sets from 1 min progressing to 4 min bouts at 90%–100% VO_2_ max or over level 18 in the Borg scale, with a safety range based on the heart reserve rate (HRR) between 60% and 90% (37). This will be interjected with active pauses at an intensity below ventilatory threshold 1 or level 12–13 on the Borg scale (safety range set at 40%–60% HRR) (38) with a duration from 3 to 5 min. When the patient is training with another device or modality, the speed or power of the exercise will be modified to achieve the intensity targets. The Borg scale (34), rather than heart rate, will be used to monitor intensity, as it is easier for the patient, who will be expected to monitor the exercise intensity on their own once they transition to home-based. Nevertheless, at the beginning of the program, the therapist will monitor patient's heart rate in parallel to ensure the equivalence with the Borg scale. The session will end with a 10 min cool-down period. The frequency of training will be 3 times per week (38).

Resistance exercises training will be performed with an elastic resistance band and a weighted bag. The recommendation will be to perform the exercises at least twice a week, and will consist of six exercises involving the major muscle groups. The load and intensity of the exercises will progress throughout the program from light to heavy elastic resistance. The intensity for each phase will be established according to the patient's rating based on the perceived exertion scale with Thera Band OMNI-Resistance exercise bands. The target is to achieve 8 to 12 repetitions of each exercise. Exercises will be performed at a cadence of 1 s for the concentric phase and 1 s for the eccentric phase. Sets for upper body exercise progress from 3 to 5 and for lower body exercise from 2 to 4. A rest period of 2 min will be allowed between sets (39).

During supervised exercise sessions, patients will be educated on how to perform exercise at home and monitor intensity, using the Borg rating of perceived exertion (RPE) or heart rate (HR). Progression will be individualized according to the progress in perceived exertion, to maintain the intensity during the whole program (36).

Over three sessions, with the first being face-to-face and subsequently via phone calls, diet management will be promoted with a nutritionist. This will include an initial evaluation, with a dietary plan agreed to prevent malnutrition and eat heart-healthily, avoid excessive salt intake (>5 g/day), improve energy and protein intake according to their exercise prescription, and achieve or maintain healthy body weight (40).

Psychosocial support and education to promote adherence to CR will be provided based on Social-cognitive theory, in which self-efficacy is a central concept (35). After an initial assessment of self-efficacy, the intervention will start with two face-to-face sessions in the first stage of the program: the first one with education about the patient's diagnosis and treatments to improve health and QoL indicators, and the second one to conduct a motivational interview (41, 42) and teach the self-monitoring behavioral change technique (43, 44). An additional third face-to-face session in the second stage will promote behavioral change through the techniques of self-control and contingency management through self-monitoring by the patient. Subsequent to these sessions and to further support the behavioral change necessary to achieve adherence to treatment, continuous communication with patients will occur with the use of mobile devices, by phone calls made by a trained collaborator. Specifically, weekly calls will be held in the third stage of the program, and every two weeks in the fourth stage (45, 46). If, during these conversations, the patient expresses the need for any supervised exercise sessions, they will be scheduled with the physiotherapist. Finally, referral to a specialist will be considered if depression is suspected upon screening.

### 2.6 Control group

The patients in the control group will receive the conventional center- and exercise-based CR at the participating centers. These programs consist of an initial assessment as well as medical and nurse management. The exercise component will comprise continuous aerobic exercise sessions plus resistance exercises. Continuous moderate exercise sessions will be of 10 min duration at the beginning of the program, and progress to 30 min by the end, as tolerated by each patient; the patient may rest between the exercises to complete the maximum tolerable exercise time. The resistance exercise training will include polyarticular and monoarticular movements against mechanical resistance or free weight. The intensity of this training will be moderate as per the Borg perceived exertion scale (34), specifically as “fairly light to somewhat hard” (score 12–13) or 50%–60% of the estimation of 1 RM. Additionally, a safety range will be set at 40%–60% HRR, which corresponds to moderate intensity. These programs typically last 10–12 weeks (3 months) with 20 supervised exercise sessions.

### 2.7 Outcomes, measures and follow-up assessments

Primary outcomes are cardiorespiratory fitness, functional capacity and HRQoL. Cardiorespiratory fitness will be assessed during a symptom-limited cardiopulmonary exercise test using an individualized gradual incremental ramp test designed to obtain oxygen consumption (VO_2_max). In brief, the VO_2_max test will consist of free-wheel pedaling with a cadence of 50–60 revolutions per minute (rpm) for 4 min on a cycle ergometer (Lode Corival, Groningen, The Netherlands), followed by 6 [New York Heart Association (NYHA) class II HF] or 10 (NYHA class III HF) watts increments every minute until the participant reaches volitional fatigue (i.e., cadence below 50 rpm or dyspnea over 8 on the Borg scale) or the emergence of test termination criteria (as extrasystoles, ST-segment elevation, arrhythmias or fibrillations on the electrocardiogram) (47). The maximal test criteria are: respiratory exchange ratio > 1.1, >85% of the age-predicted HRmax, <40 revolutions per minute, RPE >18, and plateau in VO_2_ (48). The gas exchange will be collected throughout the test using an indirect calorimeter/ergospirometer system (Cortex Metalyzer 3b (Cortex Medical, Leipzig, Germany), calibrated before the exercise test. Additionally, power output, ventilatory threshold, respiratory exchange ratio, oxygen pulse, ventilation, and respiratory rate will be assessed. HR will be recorded with a continuous telemetric HR sensor (Polar model H10, Finland) throughout the test and pos*t*-test. Additionally, if the criteria of the maximal test are not achieved, a time-limited test at 95% of the peak power output until fatigue is proposed as confirmation of the VO_2_ obtained (49). This individualized gradual incremental ramp protocol and the patient-limited nature of the test make the procedure feasible and safe even in patients with HF (12).

Functional capacity will be assessed by the six-minute walk test (6 MWT) following the American Thoracic Society Statement (50). Blood pressure, HR, oxygen saturation, and RPE will be evaluated before and immediately after the test. Patients will be instructed to walk as much as possible for 6 min. If the patients require it, breaks during the test will be allowed, but patients will be encouraged to resume walking as soon as possible. The total distance covered during the test will be recorded (meters).

HRQoL will be evaluated with the Minnesota Living with Heart Failure Questionnaire (MLHFQ) which is the most commonly used instrument for assessing HRQoL in HF patients. It contains 21 items assessing two dimensions: physical and emotional. The response options range from 0 (HRQoL unaffected) to 5 (maximum impact on HRQoL), with overall as well as dimension scores obtained from the sum of the respective items (51). This questionnaire has been validated in several languages, including Spanish (52). In addition, the generic questionnaire EQ-5D-5l will be applied, to support comparison with other health conditions and for future economic analysis of the intervention (53).

Secondary outcomes are:

– Program adherence and completion. For face-to-face activities, program adherence is defined as the percentage of total prescribed sessions completed. For home-based activities, the percentage of activities carried out at home of the prescribed ones will be computed, based on patient recordings in a diary. Program completion is considered as “yes” where participants attended most of the sessions and components of the CR intervention, and underwent a formal reassessment by the CR team at the end of the program (54).
– Pro-B-type Natriuretic Peptide (BNP), as a biomarker with prognostic utility. When it is higher than 1,000 pg/ml, there is a higher probability of having events such as hospitalizations (55, 56). The determinations will be carried out in duplicate from serum samples by the fluorescence immunoassay technique in the laboratory of one of the participating centers (Hospital Clínico de la Universidad de Chile).
– Functioning defined as the ability to perform basic, instrumental and advanced activities of daily living. It will be measured with the Activities of Daily Living Questionnaire—Technology (ADLQ-T). This instrument evaluates 7 areas related to self-care (6 items), home care and management (6 items), work and recreation (4 items), shopping and money (3 items), travel and transfers (3), communication (5 items) and technology (5). Each item is scored from 0 to 3, where 0 is no problem for the activity and 3 indicates that the activity cannot be performed. Additionally, when an aspect is part of the activities of the patient, it is scored 9. The ADLQ-T has been validated and proven useful in the Chilean population (57, 58).
– Mortality and hospital admission will be measured separately and as a composite outcome. All-cause and HF-specific mortality and hospitalization will be differentiated. Death occurrence will be monitored by study personnel through a review of a public registry. The death certificate and any associated medical documentation will be copied for consideration by the adjudicating committee. Hospitalization occurrence will be assessed by study personnel through chart review at each participating center and supplemented by phone calls to participants every two months, using a standardized script. When the participant reports a hospitalization, all associated documents (i.e., tests and exam reports) will be collected from clinical charts at each center. A central adjudication committee will review all materials. This committee will be composed of three clinician-scientists (at least one will be a cardiology specialist) who will make the final decision about the event and specify the final death cause and hospitalization diagnosis with the corresponding ICD-10 code.
– Upper and lower-body muscle strength will be assessed through grip strength and the chair stand test, respectively. Grip strength will be performed with a Jamar® Plus + electronic handheld dynamometer (Patterson Medical, Cedarburg, WI, USA) with the participant seated. Three attempts will be made on each hand alternately with 30 s of rest. The highest value of the 6 attempts will be reported (59). The chair stand test will be performed with the participant in a seated position in a chair without armrests and without wheels, feet flat on the floor, and arms crossed on the chest. From this position, they will be directed to rise fully and return to the starting position as many times as possible over 30 s. The number of repetitions achieved will be recorded (60).
– Cost will be assessed through micro-costing from the perspective of health care system. A cost pool will be constructed for each CR program. The direct cost of labor (professional and administrative), depreciation of equipment and instruments, and the goods and services of normal consumption will be included. Indirect costs will also be considered, obtained from the calculation of indirect structural costs as an average percentage of direct costs in each center. Building depreciation costs will be discounted at a factor of 0.02, a rate obtained from a previous study performed by the Chilean Ministry of Health that included all public hospitals (61). In addition, out-of-pocket spending by patients will be costed (62), as too many patients are paying out-of-pocket for CR (63) and this impacts adherence.
– Exercise-related adverse events: adverse events during exercise, such as myocardial ischemia or malignant arrhythmias, and serious adverse events, such as a death in the exercise sessions, will be reported to the corresponding ethics committee. All events will be registered for the final analysis.

All outcomes will be measured at baseline, at 3 months (equivalent to stage three in the experimental group and at the end of the program in the control group), and at 12 months of follow-up from recruitment. The personnel assessing outcomes will be blinded to intervention assignment. In Table 4, all outcomes, their measurement, and assessment schedules are presented.

**Table 4:** Outcomes, measures and assessment timing.

Outcome	Measure/source	Time point		
		Baseline	3rd month	1 year
Cardiorespiratory fitness	Symptom-limited cardiopulmonary exercise test with ergospirometer system	X	X	X
Functional capacity	6 min walk test	X	X	X
Health-related quality of life	Minnesota Living with Heart Failure Questionnaire and EuroQol-5 Dimensions.	X	X	X
Program adherence and completion	Attendance, checklist and diary.		X	
Pro-B-Type Natriuretic Peptide	Fluorescence immunoassay technique	X	X	X
Mortality and hospital admission (all-cause, cardiac-cause, and HF-cause)	Death certificate Clinical chart Medical documentation (labs, images, etc) Patient report	X	X	X
Skeletal muscle strength	Grip strength Chair stand test	X	X	X
Cost	Micro-costing method			X
Exercise-related adverse events	Checklist		X	

HF, heart failure.

In addition, sociodemographic and clinical data will be collected to compare the groups at baseline and other measures of functionality, diet, self-efficacy and perceived social support will be collected as input for the individualization of interventions.

### 2.8 Analysis plan

Baseline characteristics will be compared among the two arms of the study. This would ensure the identification of any chance imbalances despite randomization. Intention-to-treat analysis will be considered. At each follow-up point, a comparison between groups will be made. Student's *t*-tests will be used to compare the means for continuous variables (upon checking of assumptions), and confidence intervals for mean differences will be provided. For dichotomous outcomes (other than mortality), the *Z*-test for comparison of proportions will be computed along with 95% confidence intervals for the difference of proportions. To compare mortality between groups, the mortality rate ratio will be computed with 95% confidence intervals.

In case of imbalances in baseline characteristics, a multiple linear regression model will be fitted for continuous outcomes, multiple logistic regression for dichotomous outcomes, and Poisson regression (or Negative binomial) for mortality.

### 2.9 Quality control

All processes involved in the study will be overseen. The central management office will be responsible for all general processes in the study such as securing and managing ethical and regulatory approvals, document and questionnaire preparation, field personnel training, measurement standardization, and data entry monitoring. The central coordinator will be responsible for maintaining regular communication with participating centers, solving problems and supervising the overall integrity of the study.

Each center will have a local coordinator in charge of trial activities such as recruitment, random assignment, coordination of assessments, and entering data. The coordinator will be responsible for ensuring assignment concealment and blinding of outcome assessments.

Data entry will be set up on REDCap© (32), including concealed randomization and confidentiality. A dedicated person will be in charge of monitoring the data entry process, and will periodically check for completeness and integrity to resolve inconsistencies promptly.

## 3 Discussion

In response to knowledge gaps and emerging evidence regarding alternative exercise interventions and delivery models for comprehensive CR in HF, we propose the CO-CREATION-HF trial. We hypothesize that a 6-month comprehensive and hybrid model of CR, with interval exercise and self-efficacy/motivation promotion as underpinnings, will improve adherence and completion of CR, resulting in improved cardiorespiratory fitness, functional capacity, and QoL. In addition, hybrid delivery –with a 4-step transition from a typical supervised program to a patient self-administered program- may lead to lower costs for both the health system and the patient. In our model, exercise will be prescribed at the volume and intensities necessary to achieve the expected physiological effects in HF patients and patients will be supported to adhere to it.

Few clinical trials have directly evaluated the effectiveness of comprehensive, hybrid CR in HF patients, with none in low-resource settings. Two trials in high-resource settings have been seminal. First, the REACH-HF Study in patients with HFrEF (23) proved that the addition of the 12-week hybrid intervention to usual care improved HRQoL. The exercise was explicated through a manual and facilitated only at the beginning by cardiac nurses or physiotherapists. No benefit was found in other outcomes such as exercise capacity, perhaps because of patient failure to adequately engage in the exercise training program. Another trial—TELEREH-HF (24)—assessed 9-week comprehensive CR in a hybrid format (1-week center-based, plus 8 weeks home-based) leveraging technology. After 2 years of follow-up, this CR model did not result in greater survival or less hospitalization when compared to usual care, but positive effects were observed at the end of the brief intervention on functional capacity and HRQoL outcomes. An important element to consider from this trial is that the intervention exploited an advanced monitoring system with training sessions preprogrammed via tele-ECG, the latter being impractical to reproduce on a large scale or in low or middle-resource settings. A final consideration from this trial was that adherence achieved in the CR group was 88%, which may have been since patients received daily telephone contact from the center. These two trials have different and somewhat opposing approaches to patient monitoring, making it necessary to have a model that balances patient supervision and monitoring by technology with resource availability, which is crucial in low- and middle-income settings.

The main limitation of this protocol is the complex nature of the intervention to be evaluated. Although this affects generalizability and the impossibility of identifying which components potentially produce more or fewer effects, it has the advantage of testing an intervention as it is performed in practice, in addition to adhering to the recommended comprehensive nature of CR. To minimize this limitation, a detailed description of the interventions as they are carried out will be done, adhering to the best practices for intervention description using the TIDieR (Template for Intervention Description and Replication) checklist (64).

Another limitation will be the potential for contamination (i.e., that the participants in the control group participate in a comprehensive CR program). While this is a possibility given that it has been described in previous studies, in the context of this trial with an exercise-based CR control group as well as given CR in Chile is not highly comprehensive (65) and such that participants in one program would not participate in another subsequently within 9 months as there would be none proximately, it is very highly unlikely.

Finally, considering intermediate outcomes as primary outcomes, basically, maximum exercise capacity, may be an important limitation, especially for the findings to be a valid basis for potential clinical recommendations. Nevertheless, maximum exercise capacity is considered a valid predictor of such clinical events as death and hospital admission (66) and, for that reason, is the basis of our hypothesis. We have also integrated other primary outcomes, such as functional capacity as an indicator of functioning, a key outcome in CR, and HRQoL, which are equally relevant in this condition as a patient-reported outcome, and are useful for decision-making (67). Additionally, we have included the measurement of pro-BNP, a biomarker which is a good predictor of mortality and especially of hospitalization (55, 56). Likewise, and in the certainty that it is necessary to have information on the long-term impact of this type of intervention (with all its complexities), it has been planned to monitor the survival and hospitalizations of the participants beyond the 12-month follow-up.

We hypothesize that this tailored model of CR will result in greater adherence and completion of the program, resulting in improved cardiorespiratory fitness (a good predictor of mortality and hospitalization) and HRQoL in HF patients. If superiority is demonstrated, a pragmatic, hybrid (center and home-based) and comprehensive model of CR will be available for implementation in the healthcare system, especially in low and middle-income settings, to improve outcomes for the many HF patients in need.

## Ethics and dissemination

In the design of this proposal, the fulfillment of ethical principles of the Belmont Report has been considered.

Research findings will be disseminated to three main audiences. First, to reach the scientific community, they will be shared by conferences and published in peer-reviewed journals following international recommendations of the International Committee of Medical Journal Editors (ICMJE) (68). There will be two types of manuscripts of the CO-CREATION-HF: reports of the main outcomes of the study directly related to the goals and reports of data derived from CO-CREATON-HF. Second, scientific results and the corresponding CR service implications will be shared with relevant stakeholder groups (e.g., CR programs via the International Council of Cardiovascular Prevention and Rehabilitation) and policy-makers (e.g., Chile, and low-resource settings). Finally, investigators shall reach HF patients via various dissemination channels such as social media.
